# Predictive Abilities of the Frailty Phenotype and the Swiss Frailty Network and Repository Frailty Index for Non-Home Discharge and Functional Decline in Hospitalized Geriatric Patients

**DOI:** 10.14283/jfa.2022.44

**Published:** 2022-06-20

**Authors:** Anna K. Stuck, N. Schilling, D. Bertschi, A. Limacher, M. Gagesch, H. A. Bischoff-Ferrari

**Affiliations:** 1 Centre on Aging and Mobility, University Hospital Zurich and University of Zurich, c/o Stadtspital Waid, Tièchestrasse 99, 8037 Zürich, Switzerland; 2 Department of Aging Medicine, University Hospital Zurich, Zurich, Switzerland; 3 Department of Geriatrics, Inselspital, Bern University Hospital, and University of Bern, Freiburgstrasse, Bern, Switzerland; 4 CTU Bern, University of Bern, Bern, Switzerland; 5 City Hospital Zurich - Waid, University Clinic for Aging Medicine, Zurich, Switzerland

**Keywords:** Frailty syndrome, aged, geriatrics, predictive value of tests, discharge planning, inpatients

## Abstract

**Background:**

Frailty is increasingly applied as a measure to predict clinical outcomes, but data on the predictive abilities of frailty measures for non-home discharge and functional decline in acutely hospitalized geriatric patients are scarce.

**Objectives:**

The aim of this study was to investigate the predictive ability of the frailty phenotype and a frailty index currently validated as part of the ongoing Swiss Frailty Network and Repository Study based on clinical admission data for non-home discharge and functional decline in acutely hospitalized older patients.

**Design:**

Prospective cohort study.

**Setting and Participants:**

Data were analyzed from 334 consecutive hospitalized patients of a tertiary acute care geriatric inpatient clinic admitted between August 2020 and March 2021.

**Measurements:**

We assessed frailty using 1) the frailty phenotype and 2) the Swiss Frailty Network and Repository Study (SFNR) frailty index based on routinely available clinical admission data. Predictive abilities of both frailty measures were analyzed for the clinical outcomes of non-home discharge and functional decline using multivariate logistic regression models and receiver operating characteristic curves (ROC).

**Results:**

Mean age was 82.8 (SD 7.2) years and 55.4% were women. Overall, 170 (53.1%) were frail based on the frailty phenotype and 220 (65.9%) based on the frailty index. Frail patients based on the frailty phenotype were more likely to be discharged non-home (55 (32.4%) vs. 26 (17.3%); adjusted OR 2.4 (95% CI, 1.4, 5.1)). Similarly, frail patients based on the frailty index were more likely to be discharged non-home compared to non-frail patients (76 (34.6%) vs. 9 (7.9%); adjusted OR, 5.5 (95% CI, 2.6, 11.5)). Both, the frailty phenotype and the frailty index were similarly associated with functional decline (adjusted OR 2.7 (95% CI, 1.5, 4.9); adjusted OR 2.8 (95% CI 1.4, 5.5)). ROC analyses showed best discriminatory accuracy for the frailty index for non-home discharge (area under the curve 0.76).

**Conclusions:**

Frailty using the SFNR-frailty index and the frailty phenotype is a promising measure for prediction of non-home discharge and functional decline in acutely hospitalized geriatric patients. Further study is needed to define the most valid frailty measure.

**Electronic Supplementary Material:**

Supplementary material is available in the online version of this article at 10.14283/jfa.2022.44.

## Introduction

Frailty is recognized as a common geriatric syndrome associated with adverse clinical outcomes such as functional decline, hospitalization and mortality (1). The assessment of frailty has been shown to be a potentially useful prediction model in different medical settings (2). For example, frailty was shown to be associated with mortality in older patients with coronary artery disease following percutaneous coronary intervention (3). Similarly, disease outcomes were better predicted by frailty than either age or comorbidity among patients admitted to hospital with COVID-19 (4). Various approaches to the assessment of frailty have been described (5). Overall, there are two general concepts of frailty: the physical frailty phenotype and the cumulative deficit frailty (5). While the first concept described by Fried et al (6). defined frailty based on five distinct domains causing vulnerability to adverse outcomes, the second concept of deficit accumulation frailty has been operationalized into a frailty index (7) hypothesizing that the accumulation of health and functional deficits results in a decreased health state (5).

Acutely hospitalized older patients are at particular risk for adverse outcomes, such as functional decline and discharge to higher levels of care (e.g., skilled nursing facility). Consequently, in addition to treating the acute illness, care goals need to also focus on preservation or improvement of functional status to facilitate discharge to home and maintain quality of life of patients (8). Therefore, non-home discharge and functional decline are considered clinically relevant outcomes in the acutely hospitalized geriatric patients (9, 10). To identify patients at particular risk of these outcomes, clinical prediction models would be helpful to identify high risk patients for non-home discharge and functional decline upon admission and guide treatment and early discharge planning.

A promising predictor illustrated by a multitude of studies is frailty (1, 4, 11, 12). Prior studies on the role of frailty in the prediction of adverse outcomes in hospitalized patients established frailty as a risk factor for mortality (13, 14) longer hospital stay (13), and 30-day readmission (12). At the same time, Theou et al. reports in her scoping review, that a minority of studies have addressed the geriatric acute care setting (15). Further, outside the acute care setting, one prior study successfully used a frailty index based on medical records to predict hospitalization and nursing home admissions among primary care patients (16). However, to the best of our knowledge, no prior studies assessed the predictive role of frailty measures with regard to nursing home admission and functional decline in acute care (17).

The goal of this study was to investigate the predictive ability of the clinical frailty phenotype and the SFNR-frailty index under evaluation in a larger research program based on clinical admission data for non-home discharge and functional decline in acutely hospitalized older patients.

## Methods

Data were analyzed from 334 consecutive geriatric patients acutely admitted to a tertiary hospital in Bern, Switzerland, between August 2020 and March 2021. Admission criteria was age of 70 years and older, a clinical diagnosis of multimorbidity, and the need for acute inpatient care.

This is a small and preliminary subsample of the ongoing large driver project of the Swiss Frailty and Network Repository (SFNR) coordinated by the Dept. of Ageing Medicine at the University of Zurich. Selected baseline routine admission data including routinely performed baseline assessments were abstracted from patient records for each patients. Baseline assessments were performed upon admission by nurse assistants who were specifically trained for these assessments using standardized assessment tools and standardized questionnaires. In addition, selected clinical outcomes at discharge (i.e., non-home discharge, functional decline) were abstracted from hospital records for each patient. From a methodological perspective, our study is in line with a most recently published article by Oviedo et al. (18) describing sensitivity, specificity, adjusted odds ratios of different frailty measures for clinical outcome measures.

The study protocol was approved by the competent ethics committee of the Canton of Zurich (BASEC-ID 2019-00445).

### Frailty measures

We used the frailty phenotype (6) as one of the frailty measures. The frailty phenotype considers five characteristics (i.e., shrinking, low activity, fatigue, slowness, weakness) to define frailty. We used measurement protocols and cut-points for frailty as described by Gagesch et al. (19). Details on coding are summarized in the supplementary information (Table S1). Briefly, standardized questionnaires were used to investigate shrinking (loss of appetite, loose clothing, weight loss in the last 6 months), and low activity prior to admission. Weakness was assessed by grip strength, slowness by gait speed, and fatigue by the 5-item Geriatric depression scale (GDS-5). Patients who were unable to be assessed due to poor health status were assigned one frailty point for each unassessed item. Patients with a score ≥ 3 out of 5 points were considered frail.

The Swiss Frailty and Repository Consortium within the national Swiss Personalized Health Network Program developed a frailty index (FI) (19) based on the published criteria by Searle et al. (20). The validation study of the Swiss Frailty and Repository Study frailty index is ongoing and described elsewhere (19). Notably, the use of the SFNR-frailty index is preliminary in this small pilot-study and its validation is pending (19). A detailed description of the SFNR- frailty index, which consists of 30 health deficits based on the clinically routine admission data, is provided in the supplementary information (Table S2). Table S2 also details questions, assessments, coding and frequencies of each item. The SFNR-frailty index in this preliminary study was calculated for each patient by summing deficit points and dividing the sum by the total number of deficits considered. The denominator was 30 if there were no missing data. If there were missing data, the denominator was reduced by the number of missing deficits (7). Applying the definition by Kerminen et al. (21) and in accordance to a previous study in a similar setting of geriatric inpatients in Switzerland (22), patients with a total FI of ≥0.4 were considered frail. In a sensitivity analysis, we applied the cut-off of ≥0.2 for the frailty index in accordance to the study by Searle et al. (20).

### Clinical outcomes

Patients who were discharged to nursing home were classified as having a “non-home discharge”.

Functional decline at discharge was defined by the change in Barthel Index scores between admission and discharge. The Barthel Index is a 10-item ordinal scale to assess activities of daily living (ADL) in geriatric care. Items rate level of dependency in basic self-care activities (e.g., eating, dressing, bathing), sphincter controls, transfer and locomotion. Higher scores reflect a higher degree of functional independence. Functional effectiveness was defined as Barthel gain/((100 points)-(Barthel index at admission)) (22, 23). Functional decline was defined as functional effectiveness <0. This cut-off was used to conservatively separate the group of patients with a functional gain from those with functional decline. Of note, functional decline refers to the change of the overall Barthel index score (ordinal variable) from admission to discharge, whereas the frailty index includes only selected subcomponents of the Barthel, coded as dichotomous (dependent vs. independent) variables as displayed in the supplementary information (Table S2). Therefore, the independence of predictor (frailty index) and outcome (functional decline) is ensured.

Patients who died during the index hospitalization were coded as “non-home discharge” and “functional decline”, respectively. In a sensitivity analysis among surviving patients (n=325), we excluded patients from analyses who died (n=9).

### Statistical analyses

Characteristics of the study population are presented by absolute and relative frequencies or by mean and standard deviation (sd) for continuous and categorical variables. Power analysis was based on prior studies expecting a prevalence of non-home discharge to be 20% in older patients after acute geriatric hospitalization. At a two-sided confidence level of 0.05, the sample size of 334 patients yields a precision of +/−4.5% (24). Frailty instruments were correlated by using Spearman correlation coefficients. Predictive capacity (sensitivity, specificity, positive and negative predictive value) and receiver operating characteristic (ROC)-curves of frailty measures were calculated for clinical outcomes. Univariate and multivariate regression models adjusting for age and sex were calculated for each outcome and frailty measure. Sensitivity analyses were performed in subsample of 325 patients surviving hospital stay for both outcomes. Moreover, sensitivity analyses was performed applying a cut-off of ≥0.2 for definition of the frailty index. All analyzes were computed using Stata Version 16.1 (StataCorp LLC, College Station, TX, USA). An adjusted p value of <0.05 was considered statistically significant.

## Results

Overall, study patients had a mean age of 82.8 (standard deviation (sd) 7.2) years and 55.4% were women (Table 1). Median length of stay was 12 days (interquartile range (IQR) 12, 15 days).

**Table 1:** Clinical characteristics of patients (n=334)

**Demographics**	**N (%)**	**Missing, n (%)**
Age, mean (sd)	82.8 (7.2)	0
Women, n (%)	185 (55.4)	0
Weight, mean (sd)	70.8 (17.5)	1 (0.3)
Height, mean (sd)	164.5 (13.5)	0
BMI, mean (sd)	26.6 (8.7)	1 (0.3)
Length of stay, median (IQR)	15 (12, 15)	0
Summary of admission data included in the SFNR-frailty index		
Total functional score, median (IQR)	55 (40, 75)	1
5-item geriatric depression scale ≥2 points, n (%)	136 (40.7)	4 (1.2)
Ultrabrief delirium screening score:		
-Question 1 (day of the week) incorrect	67 (20.1)	0
-Question 2 (months backwards) incorrect	135 (40.4)	0
Hearing impairment, n (%)	74 (22.2)	0
Obesity (BMI ≥30kg/m2)	67 (20.1)	1 (0.3)
Pain, n (%)	46 (13.8)	46 (13.8)
Falls prior to admission	203 (60.8)	1 (0.3)
Able to climb one flight of stairs prior to admission	65 (19.5)	7 (2.1)
Walking distance <200m prior to admission	51 (15.3)	2 (0.6)
Walking aid prior to admission	183 (54.8)	0
Timed up and go test, mean (sd) (sec)	25.3 (7.5)	0
Multimorbidity, CIRS deficit score, median (IQR)	10 (8-11)	0
Decreased creatinine clearance	80 (24.0)	11 (3.3)
Hypalbuminemia	153 (45.8)	35 (10.5)
Abnormal white blood cell count	29 (8.7)	7 (2.1)
Assessments included in the clinical frailty phenotype		
Shrinking	198 (59.8)	3 (0.9)
Fatigue	127 (38.5)	4 (1.2)
Slowness	228 (70.4)^b)^	10 (3.0)
Weakness	207 (63.1)^a)^	6 (1.8)
Low activity	67 (20.1)	1 (0.3)
Frailty measures		
Clinical frailty phenotype		14 (4.2)
Score, median (IQR)	2 (2,3)	
Frail, n (%)	170 (53.1)	
SFNR-frailty index		0
Score, median (IQR)	0.46 (0.33, 0.57)	
Frail, n (%)	220 (65.9)	
Clinical outcomes		
Non-home discharge	85 (25.5)	0
Functional decline, n (%)	67 (20.1)	0

Abbreviations: sd, standard deviation; BMI, body mass index; IQR, interquartile range. a) n=51 were unable (too weak due to their underlying health condition) to perform grip strength measurement and were coded as “weak”; b) n=172 were unable (too weak to mobilize due to their underlying health condition) to perform gait speed test and were coded as “slow”

A total of 170 (53.1%) patients were classified frail based on the frailty phenotype, and 220 (65.9%) based on the preliminary use of the SFNR-frailty index (19). Proportions of the key components included in the frailty phenotype and the frailty index are listed in Table 1. Detailed description of the SFNR-frailty index, and histograms displaying the distribution of the SFNR-frailty index related to age by gender are provided in the supplementary material (Figures S1 and S2). The frailty index could be calculated in all patients (n=334), whereas the frailty phenotype could not be assessed in 14 patients (n=320) due to refusal of patients to perform assessments of the frailty phenotype (Table 1). Spearman’s rho coefficient for correlation between the frailty phenotype and the SFNR-frailty index was 0.51 (p<0.01).

Overall, 85 (25.5%) patients were not discharged home and 67 patients (20.1%) experienced functional decline. Nine (2.7%) patients died during the hospital stay. Of the nine patients who died, 7 were frail based on the frailty index and 5 were frail based on the frailty phenotype.

In univariate analyses (Table 2), the frailty phenotype and the SFNR-frailty index were both predictive for non-home discharge and functional decline. Table 2 displays the area under the curve, sensitivity, specificity, positive predictive value, negative predictive value of the frailty measures for the two clinical outcomes. Sensitivity analyses applying a cut-off of ≥0.2 for the frailty index are displayed in the supplementary information (Table S3 and S4).

**Table 2:** Predictive and discriminative capacities of the frailty phenotype and the SFNR-frailty index for clinical outcomes (n=334): Univariate analyses and AUC

**Frailty phenotype**^c)^	**OR (95%CI)**^a)^	**AUC (95% CI)**^b)^	**Sensitivity**		**Specificity**		**PPV**		**NPV**	
Non-home discharge	2.3 (1.3, 3.9)*	0.65 (0.59, 0.72)	55/81	67.9%	124/239	51.9%	55/170	32.4%	124/150	82.7%
Functional decline	2.6 (1.5, 4.8)*	0.63 (0.56, 0.70)	45/63	71.4%	132/257	51.4%	45/170	26.5%	132/150	88.0%
**SFNR-frailty index**										
Non-home discharge	6.2 (3.0, 12.8)*	0.76 (0.71, 0.82)	76/85	89.4%	105/249	42.2%	76/220	34.6%	105/114	92.1%
Functional decline	2.8 (1.4, 5.5)*	0.66 (0.59, 0.74)	55/67	82.0%	102/267	38.2%	55/220	25.0%	102/114	89.5%

Abbreviations: OR, Odds ratio; CI, confidence interval; AUC, area under the receiver operating curve; PPV, positive predictive value; NPV, negative predictive value; *p-value<0.01 for univariate logistic regression model; a) Odds ratio (95%CI) calculated from univariate logistic regression model; all frailty instruments (dependent variables) included as binary variables (frail vs. non-frail); b) AUC calculated from Receiver operating characteristic curve (ROC); frailty instruments coded as ordinal variables (clinical frailty phenotype) or continuous variable (FI); c) N=320, (n=14 missing)

Overall, 55 (32.4%) frail vs. 26 (17.3%) non-frail patients based on the frailty phenotype had a non-home discharge (adjusted OR 2.4 (95% CI, 1.4, 5.1)), whereas 76 (34.6%) frail vs. 9 (7.9%) non-frail patients based on the SFNR-frailty index had a non-home discharge (adjusted OR, 5.5 (95% CI, 2.6, 11.5)) (Table 3). Concerning functional decline, 45 (26.5%) frail vs. 18 (12%) non-frail patients based on the frailty phenotype (adjusted OR 2.7 (95% CI, 1.5, 4.9), and 55 (25.0%) frail vs. 12 (10.5%) non-frail based on the SFNR-frailty index experienced functional decline (adjusted OR, 2.8, 95% CI 1.4, 5.5). Sensitivity analyses performed in patients surviving the inhospital (n=325) stay are shown in the supplementary material (Tables S5 and S6).

Figure 1 displays the area under the curves of the frailty instruments for the outcomes non-home discharge (Panel A) and functional decline (Panel B).

**Figure 1 a:**
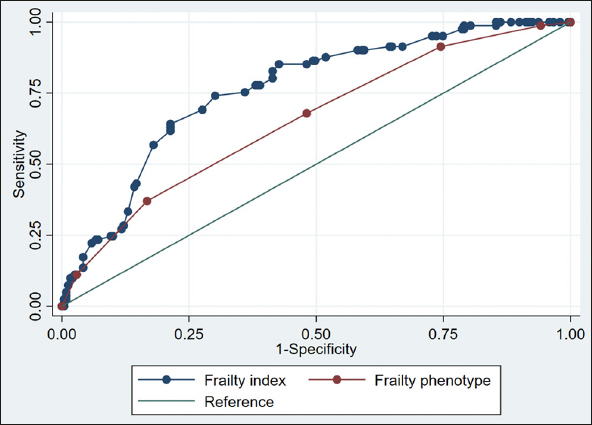
Panel A: Non-home discharge

**Figure 1 b:**
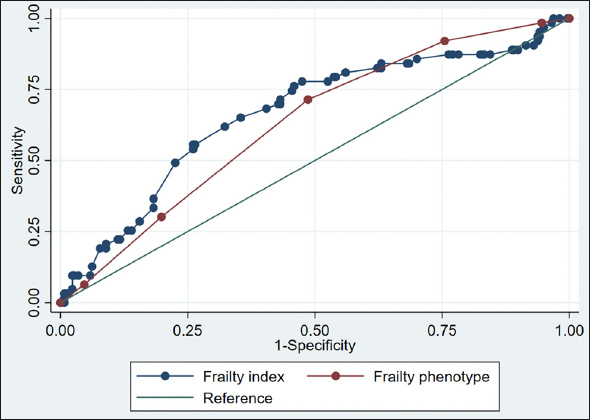
Panel B. Functional decline

## Discussion

Frailty was highly prevalent in this population of acutely hospitalized older patients using either frailty measure. We found that both the frailty phenotype and the preliminary use of the SFNR- frailty index based on regular clinical admission data significantly predicted non-home discharge, with a suggestion that the SFNR-frailty index may have a stronger discriminatory capacity for this outcome as compared to the frailty phenotype. Both measures were similarly and moderately predictive for functional decline.

To the best of our knowledge this is the first study evaluating predictive abilities of the frailty phenotype and the SFNR-frailty index based on routine clinical data to predict non-home discharge and functional decline in acutely hospitalized older patients. Prior studies either investigated different outcomes (e.g. mortality, hospitalization) (13) or investigated the same outcomes in other clinical settings (e.g. primary care) (16).

Over half of the acutely hospitalized geriatric patients in our consecutive study sample met criteria for frailty on both of the investigated frailty assessments. We explored to what extent the prevalence of frailty observed in our study is representative of the literature in acute care geriatric patients. Some studies reported very similar prevalence, such as Bieniek et al. who described a prevalence of 54.2% based on the Fried frailty phenotype in geriatric inpatients (25) or Chong et al. described a 50.0% prevalence based on the FRAIL scale (11). However, there are also studies that reported a lower prevalence, such as 19% based on a frailty phenotype defined as self-reported items of weight loss, exhaustion, slowness, weakness, and low physical activity in hospitalized medical patients aged 70 years and older (26, 27). The much lower prevalence may be explained by the fact that included patients were almost all functionally independent compared to our study population consisting of comorbid patient with a limited functional score. Moreover, the frailty phenotype in the study of Feenstra et al (26) was solely based on self-reported items possibly underestimating frailty whereas the frailty phenotype in our study included clinical measures such as gait speed and grip strength.

We also found that both the SFNR-frailty index and frailty phenotype were predictive for non-home discharge, which is in accordance to prior studies, but in other clinical settings (10, 16). In a primary care setting, an electronic frailty index using routine data to identify frailty was predictive for a combined endpoint of mortality, hospitalization and nursing home admission (16). Similarly, Sokas et al. (10) reported an association of patient-reported frailty and non-home discharge among older adults undergoing surgery. Our study adds to the literature, that both frailty tools predict non-home discharge in acute care geriatric patients.

Our relatively small study suggests a possible stronger prediction of the outcome of non-home discharge by the SNFR-frailty index. Similarly, a study assessing biological age measurements found that the frailty index along with other biomarkers (methylation age estimator GrimAge) was best predictive for mortality (28). However, our results needs further validation in a larger study. Importantly, both tools predicted non-home discharge significantly with an odds of 2.4-fold (frailty phenotype) up to 5.5-fold (SFNR-frailty index). A possible explanation for the higher discriminatory accuracy of the still to be further validated SFNR-frailty index in our study compared to the frailty phenotype might be that the SFNR-frailty index includes multimorbidity.

On the other hand, we found similar moderate predictive capacities for the frailty phenotype and the SFNR-frailty index for prediction of functional decline. Thereby, we found a good sensitivity for the frailty measure, but low sensitivities. This finding is in line with a recent study by Oviedo-Briones et al. reporting that a frailty index had a good sensitivity and a low specificity for the prediction of functional decline over one year among older patients in different clinical settings (29).

### Limitations

There are several limitations to our study. First, this is a single-site study and thus results may not apply to other settings and inpatients, or the later to be published larger SFNR study findings. Second, the preliminary use of an unvalidated frailty index needs to be interpreted with caution. Clearly, its use still needs refinement and validation, which is the main aim of the ongoing SFNR driver project coordinated by the Dept. of Aging at the University of Zurich. Third, we applied a cut-off of defining frailty based on the not yet validated new SFNR driver project frailty index and the literature-based cut-off of the frailty phenotype. Thus, we cannot exclude that other instruments or other cut-offs may reach different findings. Moreover, in a sensitivity analysis we applied the cut-off of 0.2 for defining frailty based on the frailty index. Forth, we assessed frailty using the frailty phenotype and the SFNR-frailty index. Therefore, our results cannot be extrapolated to other frailty tools such as the frailty scale (30, 31). Fifth, the inclusion of functional status items in the frailty index might lead by design to a better odds of the frailty index on functional outcomes compared to the frailty phenotype. However, the independence of the frailty index as a predictor and the outcome is maintained (the SFNR-frailty index is based on baseline data, whereas the functional outcome is based on outcome data). This methodological approach is also in accordance to another study that investigated the effect of frailty status at baseline on the effect of frailty change from admission to discharge (32). Finally, we investigated only two clinical outcomes, non-home discharge and functional decline. The larger SFNR driver project including five University Hospital sites will extend to additional clinical outcomes including length of stay, mortality and hospital readmission for both instruments.

However, even in this small study of 334 consecutive geriatric acute care patients, both frailty tools predicted both non-home discharge and functional decline. This consistent finding across both frailty tools lends support to the potential validity of the SFNR frailty index and the concept of frailty as a predictor of these two key outcomes for post-acute care planning among geriatric patients.

## Conclusions

Our data suggest that assessing frailty either by using the frailty phenotype or the SFNR-frailty index based on clinical admission data is potentially helpful for the prediction of non-home discharge and functional decline in older inpatients. In a next step, as planned in the larger SFNR driver project, these observations need confirmation and the SFNR-frailty index needs proper validation in the larger ongoing SFNR-Study.

## Electronic Supplementary Material

Appendix
