# Supplementary material for: Predictive Abilities of the Frailty Phenotype and the Swiss Frailty Network and Repository Frailty Index for Non-Home Discharge and Functional Decline in Hospitalized Geriatric Patients
Source: J Frailty Aging. 2022 Jun 20;11(4):387–92. doi: 10.14283/jfa.2022.44 (PMC9206419; doi:10.14283/jfa.2022.44)

| **Table S1. Definition of the clinical frailty phenotype** |
| --- |
| 1) Shrinking: Unintentional weight loss during last 6 months, or loss of appetite, or loose clothing, or loss of weight>5%. If the patient had a cognitive impairment (MMS ≤16 points), proxy information was collected. |
| 2) Low activity: The patient reports to engage activities that require a low or moderate level of energy such as gardening, cleaning a car, or doing a walk for “less than once a week” before admission to the hospital. If the patient had a cognitive impairment, proxy information was collected. |
| 3) Fatigue: Self-reported 5-item geriatric depression scale (5-GDS) ≥1 point. |
| 4) Slowness: <0.8m/sec measured on a standardized 4m gait speed test. Patients were asked to walk 4m (standing start, manual timing) at their usual speed. The 4-m walk test was repeated twice and patients were allowed to use an assistive device (the best of two consecutive measurements was used for analysis). If the patient had limited mobility capacity (<10m walking distance) and needed personal assistance slowness was coded as present. If a patient refused to perform gait speed testing, slowness was coded as missing. |
| 5) Weakness: Grip strength using a Martin vigorimeter at the dominant hand at the time of assessment. Three trials were performed and the maximum value achieved was used for analysis. Weakness was defined using the cut-off definitions of low grip strength according Gagesch et al. (2021). If a patient is unable to do the test due to weakness (e.g. pain, and therefore not able to press the balloon) or cognitive reason, weakness is coded as present. If a patient refused to perform grip strength testing, weakness is coded as missing. |

| **Table S2: Methodological description of the SFNR-frailty index** | | | | | | |
| --- | --- | --- | --- | --- | --- | --- |
| **Cumu-lative deficit** | **Clinical domain** | **Assessment tool** | **Cut-off point for deficit score** | **Patients with a deficit,  n (%)** | **Patients with missing values, n (%)** | **Reference** |
| 1 | Function | Barthel Index: eating | <10 points | 82 (24.6%) | 3 (0.9%) | Mahoney, F. I., & Barthel, D. W. (1965). Functional evaluation: The Barthel Index: A simple index of independence useful in scoring improvement in the rehabilitation of the chronically ill. Maryland State Medical Journal, 14, 61–65. |
| 2 |  | Barthel index: grooming | <5 points | 198 (59.3%) | 3 (0.9%) |  |
| 3 |  | Barthel index: toileting | <10 points | 194 (58.1%) | 3 (0.9%) |  |
| 4 |  | Barthel Index: transfer bed/chair | <15 points | 219 (65.6%) | 3 (0.9%) |  |
| 5 |  | Barthel index: walking | <15 points | 267 (79.9%) | 3 (0.9%) |  |
| 6 |  | Barthel index: dressing | <10 points | 274 (82.0%) | 3 (0.9%) |  |
| 7 |  | Barthel index: bowel continence | <10 points | 100 (29.9%) | 3 (0.9%) |  |
| 8 |  | Barthel index: bladder continence | <10 points | 158 (47.3%) | 3 (0.9%) |  |
| 9 | Emotion | 1) 5-item geriatric depression scale (GDS-5) | ≥2 points | 136 (40.7%) | 4 (1.2%) | Hoyl TM, Alessi CA, et al. Development and Testing of a five-item version of the Geriatric depression scale. J Am Geriatr Soc 1999; 47: 873-878. |
| 10 | Cognition | Ultrabrief delirium screening: Question 1: “What day of the week is it?” | Incorrect answer | 67 (20.1%) | 0 (0%) | Fick DM, Inouye SK, Guess J, Ngo LH, Jones RN, Saczynski JS, Marcantonio ER. Preliminary development of an ultrabrief two-item bedside test for delirium. J Hosp Med. 2015 Oct;10(10):645-50. |
| 11 |  | Ultrabrief delirium screening:  Question 2: «Months of the year backwards?” | Incorrect answer | 135 (40.4%) | 0 (0%) |  |
| 12 | Hearing | Best corrected hearing using whisper test (with hearing aid if present) | <2/3 | 74 (22.2%) | 1 (0.3%) | Bagai A, Thavendiranathan P, Detsky AS. Does this patient have hearing impairment? JAMA 2006;295:416-28 |
| 13 | Nutrition | Body mass index: Overweight | BMI ≥30kg/m^2^ | 67 (20.1%) | 0 (0%) | According to definition by the World Health Organization |
| 14 |  | Grip strength | Low grip strength according to reference cut-off values | 143 (42.8%) | 6 (1.8%) | Gagesch M, Abderhalden L, Kressig RW, et al. Threshold definition for grip strength to identify 267 relevant weakness in Swiss DO-HEALTH participants. International Conference on Frailty and 268 Sarcopenia Research; 2019; Miami Beach, FL, USA. |
| 15 | Pain | Numeric rating scale upon admission | >3 points | 46 (13.8%) | 46 (13.8%) | Boonstra AM, Stewart RE, Köke AJ, Oosterwijk RF, Swaan JL, Schreurs KM, Schiphorst Preuper HR. Cut-Off Points for Mild, Moderate, and Severe Pain on the Numeric Rating Scale for Pain in Patients with Chronic Musculoskeletal Pain: Variability and Influence of Sex and Catastrophizing. Front Psychol. 2016 Sep 30;7:1466. |
| 16 | Mobility | Falls in the last 12 months | Yes | 203 (60.8%) | 1 (0.3%) | Bandeen-Roche K, Seplaki CL, Huang J, Buta B, Kalyani RR, Varadhan R, Xue QL, Walston JD, Kasper JD. Frailty in Older Adults: A Nationally Representative Profile in the United States. J Gerontol A Biol Sci Med Sci. 2015 Nov;70(11):1427-34. |
| 17 |  | Stairs: Able to climb 1 flight of stairs (10 steps) prior to hospital admission | No, or personal assistance needed | 65 (19.5%) | 7 (2.1%) | Bandeen-Roche K, Seplaki CL, Huang J, Buta B, Kalyani RR, Varadhan R, Xue QL, Walston JD, Kasper JD. Frailty in Older Adults: A Nationally Representative Profile in the United States. J Gerontol A Biol Sci Med Sci. 2015 Nov;70(11):1427-34. |
| 18 |  | Walking distance: Able to walk 200m prior to hospital admission | No, or personal assistance needed | 51 (15.3%) | 2 (0.6%) | Shumway-Cook A, Ciol MA, Yorkston KM, Hoffman JM, Chan L. Mobility limitations in the Medicare population: prevalence and sociodemographic and clinical correlates. J Am Geriatr Soc. 2005 Jul;53(7):1217-21. doi: 10.1111/j.1532-5415.2005.53372.x. PMID: 16108942. |
| 19 |  | Walking aid: Use of walking aid prior to hospital admission | Yes (walking stick(s), walking frame with or without wheels, wheelchair) | 183 (54.8%) | 0 (0%) | Shumway-Cook A, Ciol MA, Yorkston KM, Hoffman JM, Chan L. Mobility limitations in the Medicare population: prevalence and sociodemographic and clinical correlates. J Am Geriatr Soc. 2005 Jul;53(7):1217-21. doi: 10.1111/j.1532-5415.2005.53372.x. PMID: 16108942. |
| 20 |  | Timed up and go (TUG) | TUG ≥20sec, inability to mobilize and/or personal assistance needed | 249 (74.6%) | 0 (0%) | Podsiadlo D, Richardson S. The timed “up & go”: a test of basic functional mobility for frail elderly persons. J Am Geriatr Soc. 1991;39:142–8. |
| 21 | Multimorbidity | Cumulative illness rating scale (CIRS): CIRS: Respiratory | ≥1 point per item | 158 (47.3%) | 0 (0%) | Fabio Salvi, MD, Mark D. Miller, MD, Adele L. Towers, MD, Valeria Morichi, MD, and Paolo Dessı-Fulgheri, MD; Guidelines for Scoring the Modified Cumulative Illness Rating Scale; J Am Geriatr Soc. 2008 Oct;56(10) |
| 22 |  | CIRS: Upper gastrointestinal |  | 136 (40.7%) | 0 (0%) |  |
| 23 |  | CIRS: Lower gastrointestinal |  | 235 (70.4%) | 0 (0%) |  |
| 24 |  | CIRS: Hepatic and pancreatic |  | 110 (32.9%) | 0 (0%) |  |
| 25 |  | CIRS: Renal |  | 143 (42.8%) | 0 (0%) |  |
| 26 |  | CIRS: Genitourinary |  | 230 (68.9%) | 0 (0%) |  |
| 27 |  | CIRS: Neurological |  | 229 (68.6%) | 0 (0%) |  |
| 28 | Laboratory values | Decreased creatinine clearance | Creatinine ≥104umol/l | 80 (24.0%) | 11 (3.3%) | Cut-off according to local laboratory |
| 29 |  | Abnormal white blood cell count | WBC: >10.5 or <3.0 G/l | 29 (8.7%) | 7 (2.1%) | Cut-off according to local laboratory |
| 30 |  | Hypalbuminemia | Albumin <35G/l | 153 (45.8%) | 35 (10.5%) | Cut-off according to local laboratory |

| **Table S3. Sensitivity analyses: Predictive and discriminative capacity of the SFNR-frailty index using a cut-off of 0.2 (n=334):  Univariate analyses and AUC** | | | | | | | | | | |
| --- | --- | --- | --- | --- | --- | --- | --- | --- | --- | --- |
| Frailty index | OR (95%CI) ^a)^ | AUC (95% CI) ^b)^ | Sensitivity |  | Specificity |  | PPV |  | NPV |  |
| Non-home discharge | n.a. ^c)^ | 0.76 (0.71, 0.82) | 85/85 | 100% | 21/249 | 8.4% | 85/313 | 27.2% | 21/21 | 100% |
| Functional decline | 0.79 (0.28, 2.2) | 0.66 (0.59, 0.74) | 62/67 | 92.5% | 16/267 | 6.0% | 62/251 | 80.2% | 16/21 | 76.2% |

*Abbreviations: OR, Odds ratio; CI, confidence interval; AUC, area under the receiver operating curve; PPV, positive predictive value; NPV, negative predictive value; n.a., not applicable*

*p-value<0.01 for univariate logistic regression model

1. Odds ratio (95%CI) calculated from univariate logistic regression model; all frailty instruments (dependent variables) included as binary variables (frail vs. non-frail)
2. AUC calculated from Receiver operating characteristic curve (ROC); frailty instruments coded as ordinal variables (clinical frailty phenotype) or continuous variable (FI)
3. frailty index in the univariate model omitted, as frailty index using cut-off 0.2 predicts non-home discharge perfectly

| **Table S4. Sensitivity analyses: Predictive capacity of the SFNR-frailty index using a cut-point of 0.2 for clinical outcomes: Multivariate analyses (n=334)** | | | |
| --- | --- | --- | --- |
|  | Frailty index ^b)^ | |  |
|  | Adjusted OR ^a)^ (95% CI) | p-value |  |
| Non-home discharge | n.a.^c)^ | n.a.^c)^ |  |
| Functional decline | 0.73 (0.25, 2.10) | 0.560 |  |

*Abbreviations: OR, Odds ratio; CI, confidence interval; n.a., not applicable*

1. multivariate logistic model adjusted for age and sex
2. all frailty instruments (dependent variables) included as binary variables (frail vs. non-frail)
3. frailty index in the multivariate model omitted, as frailty index using cut-off 0.2 predicts non-home discharge perfectly

| **Table S5. Sensitivity analyses: Predictive and discriminative capacities of the clinical frailty phenotype and the SFNR-frailty index for clinical outcomes in surviving patients (n=325): Univariate analyses and AUC** | | | | | | | | | | |
| --- | --- | --- | --- | --- | --- | --- | --- | --- | --- | --- |
| Clinical frailty phenotype ^c)^ | OR (95%CI) ^a)^ | AUC (95% CI) ^b)^ | Sensitivity | | Specificity | | PPV |  | NPV |  |
| Non-home discharge | 2.2 (1.3, 3.9)* | 0.65 (0.58, 0.72) | 50/74 | 67.7% | 124/239 | 51.9% | 50/165 | 30.3% | 124/148 | 83.8% |
| Functional decline | 2.5 (1.4, 4.7)* | 0.61 (0.54, 0.68) | 41/58 | 70.7% | 131/255 | 51.4% | 41/165 | 24.9% | 131/148 | 88.5% |
| Frailty index |  |  |  |  |  |  |  |  |  |  |
| Non-home discharge | 6.3 (2.7, 14.3)* | 0.77 (0.71, 0.83) | 69/76 | 90.8% | 105/249 | 42.2% | 69/213 | 32.4% | 105/112 | 93.8% |
| Functional decline | 2.7 (1.4, 5.5)* | 0.65 (0.57, 0.73) | 49/60 | 81.7% | 101/265 | 38.1% | 49/213 | 23.0% | 101/112 | 90.2% |

*Abbreviations: OR, Odds ratio; CI, confidence interval; AUC, area under the receiver operating curve; PPV, positive predictive value; NPV, negative predictive value;*

*p-value<0.01 for univariate logistic regression model

1. Odds ratio (95%CI) calculated from univariate logistic regression model; all frailty instruments (dependent variables) included as binary variables (frail vs. non-frai
2. AUC calculated from Receiver operating characteristic curve (ROC); frailty instruments coded as ordinal variables (clinical frailty phenotype) or continuous variable (FI)
3. N=313, (n=7 missing)

| **Table S6. Sensitivity analyses: Predictive abilities of the clinical frailty phenotype and the SFNR-frailty index for clinical outcomes in surviving patients: Multivariate analyses (n=325)** | | | | | |
| --- | --- | --- | --- | --- | --- |
|  | Clinical frailty phenotype ^b) c)^ | | Frailty index ^b)^ | |  |
|  | Adjusted OR ^a)^ (95% CI) | p-value | Adjusted OR ^a)^ (95% CI) | p-value |  |
| Non-home discharge | 2.4 (1.3, 4.2) | 0.003 | 6.3 (2.7, 14.3) | <0.001 |  |
| Functional decline | 2.6 (1.4, 4.8) | 0.003 | 2.7 (1.3, 5.5) | 0.006 |  |

*Abbreviations: OR, Odds ratio; CI, confidence interval;*

1. multivariate logistic model adjusted for age and sex
2. all frailty instruments (dependent variables) included as binary variables (frail vs. non-frail)
3. N=320, (n=14 missing)

**Figure S1.**

**Panel A. Kernel density histogram of the SFNR-frailty index in women**


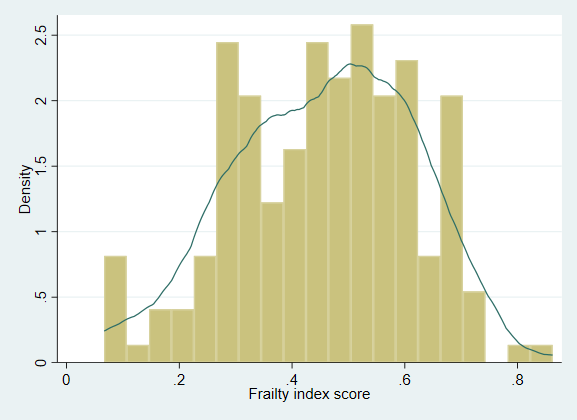


**Panel B. Histogram of the SFNR-frailty index in men**


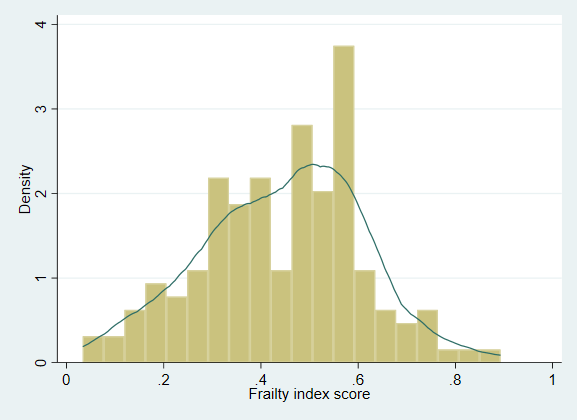


**Figure S2. Scatterplot of the SFNR-frailty index and age by gender**


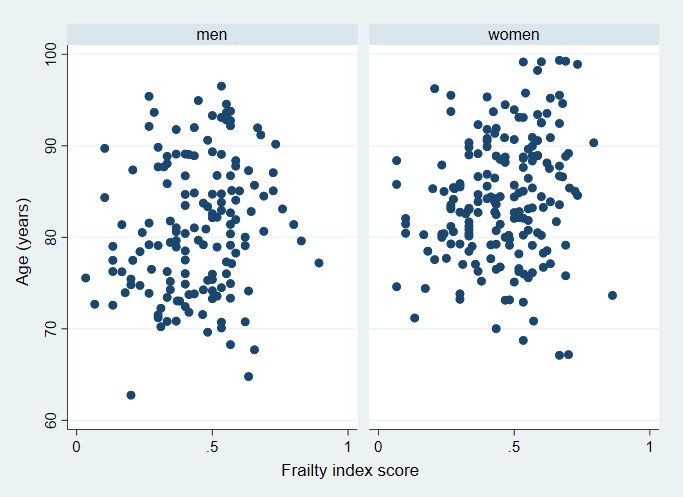

Supplement: Supplementary file 1 — Appendix [file 42415_2022_44_MOESM1_ESM.docx]
